# A critical review of medicolegal research and information asymmetries in investigating cases of extrajudicial executions and forced disappearances

**DOI:** 10.1007/s00414-025-03411-7

**Published:** 2025-02-03

**Authors:** Nicholas Dempsey, Reena Sarkar, Claudia Rivera, Richard Bassed

**Affiliations:** 1 Department of Forensic Medicine, Monash University, 65 Kavanagh Street, Southbank, VIC 3006 Australia; 2 Department of Forensic Anthropology, The Guatemalan Forensic Anthropology Foundation, Guatemala City, Guatemala; 3 Victorian Institute of Forensic Medicine, Kavanagh Street, Southbank, VIC 3006 Australia

**Keywords:** Extrajudicial execution, Forced disappearance, Skeletal trauma, Torture, Mass grave, Information asymmetry

## Abstract

Medicolegal systems investigate the cause and manner of death, particularly differentiating between unintentional and intentional deaths. The examination of remains from unlawfully killed individuals is critical in exposing human rights violations. However, forensic medical investigations of these human remains can face multiple challenges, especially in contexts marked by limited resources, political influence, and sub-optimal investigative procedures. When killings are state-sanctioned or facilitated by well-resourced non-state actors, the clandestine disposal of remains can create a culture of impunity, leaving affected families and communities without recourse or resolution. This study aggregates articles in English and Spanish, examining the current state of how forensic medical research on extrajudicial executions and forced disappearances informs practice. It highlights critical gaps in the empirical literature, particularly in the reporting of the scientific findings that impact the investigation of victims of these unlawful killings. These cases' inherently non-linear and unpredictable nature, often influenced by chaotic and unstable conditions, can create disproportionate challenges for forensics practitioners. To address these gaps, this review suggests leveraging epidemiological frameworks to track data trends in these unlawful killings, supporting public health initiatives in prevention and policy. It emphasises the need for comprehensive documentation, robust databases, and adaptive forensic methodologies to navigate uncertainties and systemic limitations inherent in this complex and unpredictable domain of medicolegal death investigation.

## Introduction

Death investigations for suspected victims of extrajudicial executions and forced disappearances are complex. Forensic practitioners who work in humanitarian contexts appreciate that these deaths may present significant practical and legal challenges [1]. In such cases, the state is obligated to investigate; however, many states tacitly or explicitly fail to prevent violations of the right to life [2]. These unlawful killings can often follow a sequence of events—capture, detention, and disposal [3] – each phase featuring unique uncertainties and investigative challenges. For example, signs of healing or healed fractures may indicate prolonged exposure to abuse, providing critical insights into the sequence of events and the victim’s experience and helping clarify some of the circumstances of these types of deaths [4–6]. However, specific empirical research about extrajudicial executions and forced disappearances is limited [7].

This is understandable given the clandestine circumstances of these deaths, which can create significant challenges in accessing human remains, as well as access to legal, operational and statistical data [8]. Consequently, as remains decompose, identification, trauma analysis and determination of cause/manner of death may be impossible [9, 10]. The circumstances of these deaths are often obscured by complex sociopolitical factors, creating significant issues for investigators [11]. This unpredictability and uncertainty, combined with limited data (and hidden variables), can distort conclusions about these deaths and lead to erroneous investigation decision-making [12, 13], underscoring the need for caution when interpreting these events retrospectively [14]. During times of political or social instability, resources for death investigations may be limited, suboptimal or absent, forcing improvised methods for managing the dead and the missing [7, 15]. Societal disruptions can cause medicolegal death investigation systems to be overwhelmed, delayed or prevented in conducting investigations [16], exposing the fragility of systems under stress [17] and potentially resulting in substandard responses and investigative outcomes. The situation is further complicated as many victims of extrajudicial killings or forced disappearances most often belong to communities repeatedly subjected to inequalities, which can result in a lack of reliable antemortem records [18]. Besides ensuring state accountability, the investigation of these deaths can foster fairness and permit families and societies to grieve and heal [19, 20]. In some regions, along with conventional death investigation systems, International Non-Government Organisations (INGOs) may actively deliver search and recovery services. A lack of standardised operating procedures, resources, mandates, and the involvement of multiple organisations can often result in a chaotic corpus of information that complicates or even prevents investigation and the safe return of remains to families [15, 21]. For example, mass deaths associated with migratory sea routes [22], sectarian violence and ethnic cleansing highlight topical issues such as a lack of international agreement, local political contexts hindering victim analysis, and a lack of resources. This review will solely focus on the forensic medical aspects of extrajudicial killings and forced disappearances.

The review aims to 1) establish the status of research on extrajudicial killings and forced disappearances and 2) highlight how research impacts forensic practice regarding these types of killings. This review acknowledges the limited availability of empirical literature reporting pathological, anthropological, scientific, and other investigative findings within this area of forensic practice. It emphasises the need to recognise how limited data can affect the capacity for robust analysis to influence policy and prevention effectively. By examining the patterns and prevalence of trauma associated with extrajudicial executions and forced disappearances while accounting for data constraints, this review advocates for a cautious, adaptive approach to address the uncertainty and issues of data scarcity in the investigation of extrajudicial killings and forced disappearances.

## Methodology

**Fig. 1:**
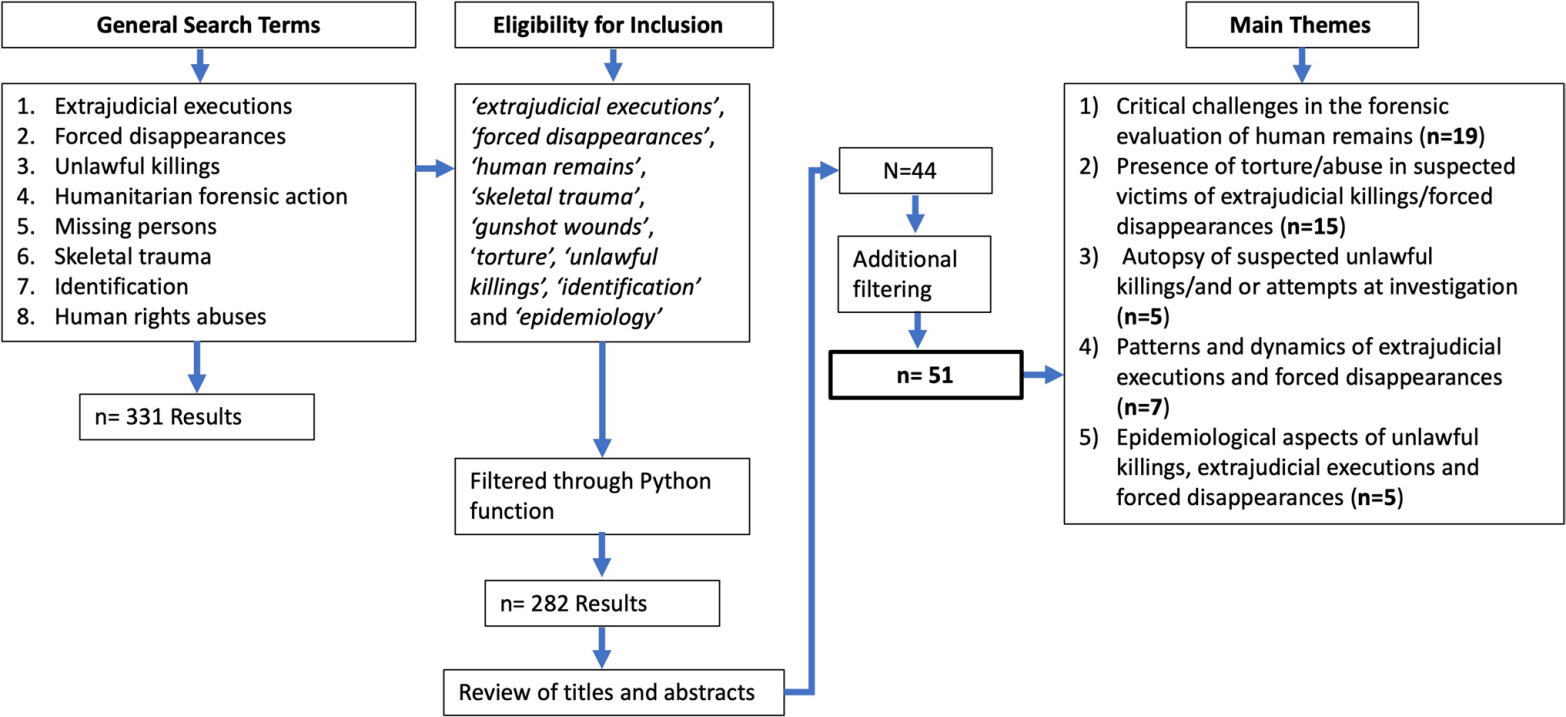
Literature review search protocols and results through the inclusion and filter process

To ensure that the most relevant publications were found, a web scraper using Beautiful Soup [23] (an HTML parser) was programmed using the Python coding language to collate articles. This obtained publications through Google Scholar (Elsevier, Springer, Wiley, Taylor and Francis, Sage, etc.). These methods have been used successfully in an independent study on the analysis of skeletal trauma in medicolegal contexts [24]. This method was designed to gather pertinent information such as article titles, publication years, citation counts, and publication links, which could also be scanned for relevancy. The scraper returned articles based on broad search terms *‘extrajudicial executions’*, *‘forced disappearances’, ‘unlawful killings’*, ‘*humanitarian forensic action’, ‘missing persons’, ‘skeletal trauma’, ‘identification’*, and *‘human rights abuses’*. These were mapped to specific search terms outlined in Fig. 1**,** detailing the search protocol. Each of these search term results was collated and saved as a.CSV file containing the paper's title, year, number of citations, and link to the publication—the three separate.CSV files were collated into a single master copy and imported into Python as a Pandas [25] data frame allowing analysis of the results and removal of duplicate papers. This was done for both English and Spanish documents. For the remaining publications, the inclusion criteria required keywords; *‘extrajudicial executions’*, *‘forced disappearances’*, *‘human remains’*, *‘skeletal trauma’*, *‘gunshot wounds’*, ‘*torture’, ‘unlawful killings’, ‘identification’* and *‘epidemiology’* in their titles and truncated abstracts, saved to a Python dictionary for additional filtering. A Python function was used to filter these terms from the publications, which were compiled into the final data frame and saved as an Excel spreadsheet with embedded links that allowed the review of the abstracts to align with the aims of this review. Publications that did not include any of the above terms were excluded. No restrictions were placed on the date of the publication. The review considered study types, including reviews, empirical research and case reports. Topics included trauma analysis of human remains, torture, executions, fatal injury descriptions, recovery and identification of remains, and medicolegal investigations. Materials from conference abstracts, government reports, UN/UNDOC reports, and publications describing legislative interventions and legal cases were also excluded. To identify themes in the publications, topic modelling (natural language processing technique) was applied using the Natural Language Toolkit [26]. The final publications included and discussed in this review focus on the available literature on extrajudicial executions and forced disappearances to best provide a forensic science/forensic medicine perspective on these types of killings.

## Results

The methodology initially yielded a total of *n* = 331 articles. *N* = 282 articles were excluded as they were duplicates or irrelevant using a keyword-reliant filtering function (see Fig. 1). When filtered with specific keywords, *n* = 44 articles remained. However, further review of this content increased the total to *n* = 51 as several publications also had overlapping themes. The main overall themes were:

1. *Critical challenges in the forensic evaluation of human remains (n* = *19).*
2. *Presence of torture/abuse in suspected victims of extrajudicial killings/forced disappearances (n* = *15).*
3. Autopsy of suspected extrajudicial executions/forced disappearances and or attempts at investigation. *(n* = *5).*
4. *Patterns and dynamics of extrajudicial executions and forced disappearances (n* = *7).*
5. *Epidemiological aspects of unlawful killings, extrajudicial executions and forced disappearances (n* = *5).*

The literature reviewed included *n* = 11 literature reviews or book chapters, *n* = 8 case reports, *n* = 14 empirical or observational studies, and *n* = 11 exploratory or descriptive studies. Details of the publications are presented in Table 1..

**Table 1:** Classification of publications by study types, trauma indicators, and medicolegal death investigations*

Publication	Study Type				Trauma indicators in human remains, and medicolegal death investigation			
	Literature reviews/book chapters/books/commentary pieces (*n* = 11)	Case reports/case studies (*n* = 8)	Empirical/Observational studies (*n* = 14)	Exploratory/Descriptive studies (*n* = 11)	Analysis of trauma/injury patterns	Signs of torture/and or abuse	Signs of execution (extrajudicial/judicial)	Medicolegal death investigation/ recovery and identification of human remains
Large-scale forensic investigations into the missing: Challenges and considerations [16]	×	×	×		×	×	×	
Missing Persons Investigations and Identification: Issues of Scale, Infrastructure, and Political Will [9]		×	×	×	×	×	×	
Understanding Mortality Patterns in Complex Humanitarian Emergencies [27]	×	×		×	NA	NA	NA	NA
Civilian killings and disappearances during civil war in El Salvador (1980–1992) [28]	×	×		×	NA	NA	NA	NA
Violent deaths of Iraqi civilians, 2003–2008: analysis by perpetrator, weapon, time, and location [29]	×	×	×		×			×
War and mortality in Kosovo, 1998–99: an epidemiological testimony [30]	×	×		×		×	×	×
Muertes en conflicto: aproximación epidemiológica descriptiva a una muestra de presuntas ejecuciones extrajudiciales ocurridas en Colombia en el período 2002–2012 [31]	×	×		×		×		×
The pathology of torture [32]		×	×	×			×	
Evidence of multiple methods of torture in a case from Sri Lanka [33]	×		×	×	×		×	×
Bone trauma [10] (in Encyclopedia of Global Archaeology)		×	×	×			×	×
Trauma patterns in cases of extrajudicial executions [34]	×	×	×	×		×	×	
Skeletal Trauma: identification of injuries resulting from human rights abuses and armed conflict [35]		×	×	×				
Variation of gunshot injury patterns in mortality associated with human rights abuses and armed conflict: an exploratory study [36]	×	×	×			×	×	×
Marks of autopsy and identification of victims of human rights violations exhumed from cemeteries: the case of the Spanish Civil War (1936–1939) [37]	×	×	×					
Forensic Anthropology in Investigations of Crimes Against Humanity: Global Dimensions and the Mid-19th-Century Ajnala (India) Massacre [38]	×		×	×			×	×
Timoteo Mendieta Alcalá and the Pact of Forgetting: trauma analysis of execution victims from a Spanish Civil War mass burial site at Guadalajara, Castilla la Mancha [39]	×	×		×				×
The possibility of establishing causes of death on the basis of the exhumed remains of prisoners executed during the communist regime in Poland: the exhumations at Powązki Military Cemetery in Warsaw [40]	×	×		×				×
Gunshot wounds (resulting from execution) of exhumed victims of the communist regime in Poland [41]	×		×	×		×		×
Skeletal evidence of the ethnic cleansing actions in the Free City of Danzig (1939–1942) based on the KL Stutthof victims analysis [42]	×		×	×				×
Analysis of skeletal trauma on the bodies found in a mass grave [43]	×	×	×			×		×
The examination and reporting of war crimes—an example from Finnish history [44]	×	×		×	×			
Intersite analysis of victims of extra- and judicial execution in civil war Spain: Location and direction of perimortem gunshot trauma [45]	×	×		×		×		×
A potential contribution to human identification using peri-mortem trauma: An example from Peru [46]	×	×		×		×		×
Radiology of Torture [47]	×	×	×				×	×
Osteological proofs of torture and cruelty: forensic findings form a secret cemetery in Tirana, Albania [48]	×	×		×				×
Historical autopsy: A contribution to the investigation of State terrorism crimes in Uruguay [49]	×		×	×		×	×	×
The challenges presented by decomposition [50]	×		×		×		×	×
Epidemiology of Genocide: An Example from the Former Yugoslavia [51]	×	×		×	×	×	×	
Multidisciplinary Forensic Approach in “Complex” Bodies: Systematic Review and Procedural Proposal [52]		×	×	×		×	×	
Assessing wound vitality in decomposed bodies: a review of the literature [53]		×	×	×		×	×	
Percentage of Body Recovered and Its Effect on Identification Rates and Cause and Manner of Death Determination [54]	×	×		×		×	×	
Forensic Anthropology and the Most Probable Cause of Death in Cases of Violations Against International Humanitarian Law: An Example from Bosnia and Herzegovina [54]	×	×		×		×	×	
Patterns of Trauma in Conflict Victims from Timor Leste [55]	×	×		×		×	×	
The importance of increasing the forensic relevance of oral health records for improved human identification outcomes [56]	×	×	×		×	×	×	
Antemortem dental records versus individual identification [57]	×	×	×		×	×	×	
Dignification of Victims Through Exhumations in Colombia [58]	×	×	×		×	×	×	
Role of forensic odontology in the identification of victims of major mass disasters across the world: A systematic review [59]		×	×	×	×	×	×	
Postmortem Criminal Mutilation in Panama [60]	×		×	×			×	×
The investigation of the human remains of the "disappeared" in Argentina [61]		×	×	×	×	×	×	
Skeletal Evidence for Child Abuse: A Physical Anthropological Perspective [62]		×	×	×			×	
The role of the pathologist in human rights abuses [63]		×	×	×				
Confirmation of alleged falanga torture by bone scintigraphy—Case report [64]	×		×	×			×	
Identification and Differential Diagnosis of Traumatic Lesions of the Skeleton [65]		×	×	×			×	×

*Many studies had overlapping categories, but the above table features only the predominant study type

### Critical challenges in the forensic evaluation of human remains/and investigation

*N* = 19 papers highlighted critical challenges in the forensic evaluation of human remains and their investigation. These studies detailed complications in the analysis of human remains due to decomposition [50] and commingling, as well as how these factors hinder the assessment of features [50] suggestive of the circumstance and cause of death [16, 52, 53, 66]. Komar and Potter [54] analysed *n* = 773 forensic anthropology cases. They noted that when bodies were complete, estimating the circumstance and cause of death was between 83–79%, but declined to 40% when less than half of the body was available. At the Powązki Military Cemetery in Warsaw, *n* = 194 executed prisoners were exhumed, whereas, in *n* = 86 cases, it was not possible to establish the cause of death due to poor preservation of the remains [40]. In Timor Leste, trauma data from the autopsies and anthropological reports of *n* = 105 victims from the 1999 conflict showed that decomposition and the percentage of the body recovered significantly impacted the presence or absence of trauma, where complete remains were 10.4% more likely to show signs of trauma [55].

Furthering this issue, other studies discuss how osteological lesions may not fully account for the traumatic impacts on a body, given bones are resistant to impact [39]. Furthermore, injury to the skeleton is insufficient to establish motive, intent, and perpetrator identity [36, 43]. Other publications noted that identification rates from mass killings are low [67], attributed to the condition of the remains, absence of antemortem records [56, 57], technological issues, economics, and political will [9, 58, 68]. One forensic odontology study on identification reviewed the research question regarding accessibility to antemortem records and how a damaged infrastructure can affect successful identification [59], demonstrating that random events can affect reliable recordkeeping.

### Signs of torture/abuse in suspected victims of extrajudicial killings/forced disappearances

*N* = 15 studies examined torture, documenting how victims can exhibit signs of healed/healing injury, indicating beatings before death and disposal [10, 32, 33, 48, 68] and how dismemberment of hands, feet and teeth (wholly or partially missing) [60] interfered with identification efforts [61]. Other publications demonstrated how antemortem trauma can be indicative of chronic abuse [62] attributed to the duration spent in custody, allowing injuries to heal [63], a characteristic of these cases [69]. Some studies discussed specific types of torture and potential visibility in skeletal tissue of the effects of this abuse, such as ‘Falanga’ or ‘Falaka’ (beating of the soles of the feet) [47]. Rodríguez-Martín discussed identification and differential diagnosis of traumatic skeletal injuries [65], noting suspension torture and its effect on the skeleton, including dislocation of the glenohumeral joint when an individual is suspended from their backwards-bent arms. Another study detailed how bone scintigraphy can reveal increased blood flow to the feet and distal tibia [64] (as well as in circumstances where there are rib microfractures [33]), potentially indicating torture. In Guadalajara (Castilla la Mancha), Spain, out of *n* = 28 executed individuals, *n* = 5 showed signs of healing injuries, potentially indicative of assaults leading up to execution, with one individual having sustained 27 fractures [39]. In another example, *n* = 109 individuals analysed from the 1986 Pedro-Lurigancho prison massacre in Lima, Peru, revealed a subgroup of *n* = 4 individuals with gunshot wounds (GSW) and blunt force trauma (BFT) injuries, suggesting targeted violence [46].

### Autopsy of suspected extrajudicial executions/forced disappearances and or attempts at investigation

*N* = 5 studies described autopsy results in the context of suspected unlawful killings/and or attempts at investigation. One report highlighted issues when autopsies produced multiple results ranging from “suicide from jumping from a window” and “gunshot injury to the head with the body being staged as if it had fallen through a window” [34]. Another documented autopsies in suspected torture of victims [32]. In another study, *n* = 19 autopsies were conducted on extrajudicial killing victims from the Spanish Civil War, indicating that judicial processes to investigate suspected violent criminal deaths were initially performed in the early phase of the conflict [37]. An examination of historical autopsy reports demonstrated the extent of coverups in official versions and may imply criminal responsibility of the State [49]. The Uruguayan judicial admitted this evidence, proving the falsity of official state narratives [49]. In one study documenting institutional failures [44], original conclusions about the killings were incorrect due to incorrect postmortems that were potentially the result of political influence.

### Patterns and dynamics of extrajudicial executions and forced disappearances

*N* = 7 studies discuss the patterns and dynamics of extrajudicial killings and forced disappearances. One study examined victims of the Spanish Civil War, analysing their lives until execution, comparing rural and cemetery extrajudicial cases where GSW to the skull was oriented predominantly back to front (70.7% and 61%) compared to judicial GSW cases, which were with 70.7% front to back, [39]. Another study showed variations in GSW patterns associated with human rights abuses, with discrete areas of the body and centre of mass preferred over extremities [36]. In another, evidence of overkill (injuries that exceed what is necessary to kill a person) or a lack of defensive wounds may demonstrate brutality in the execution process, exceeding state-sponsored execution protocols [42]. This was similarly reported in another study where skeletal evidence demonstrated that execution methods contravened judicial execution protocol [41]. Results from a Mid-19th-Century Massacre in Ajnala, India, showed that *n* = 282 soldiers from a rebellion were under restraint when then shot in the back of the head, demonstrating extrajudicial killings [38]. Another paper examined *n* = 183 potential cases of extrajudicial execution, statistically evaluating the probability that death could be classified as extrajudicial using principle component analysis (PCA) [31]. An inter-site study comparing different locations identified differences in the distribution of cranial GSW between judicial and extrajudicial execution sites [45].

### Epidemiological aspects of unlawful killings, extrajudicial executions and forced disappearances

*N* = 5 studies looked at population-based aspects of unlawful killings. These studies included modelling to estimate the number of unaccounted cases [29], analysing individuals killed at Srebrenica for pathologies indicative of victims’ health status to provide policymakers’ risk assessments to use in their assessments for genocide prevention [51], and examining mortality rates in humanitarian situations due to unlawful killings [27], forced disappearances [28]; violent deaths [29] and risk of mortality from war-related trauma [30].

## Discussion

This review assessed how forensic medical research on extrajudicial executions and forced disappearances informs practice. It highlighted critical gaps in the empirical literature, particularly in reporting the pathological, anthropological, and other scientific findings. Given the constraints on accessing sufficient data, a cautious approach that acknowledges these systemic limitations is necessary. Information asymmetries heighten the uncertainties and vulnerabilities [70] of forensic investigations, rendering them more susceptible to errors and less robust in situations involving incomplete or biased data. The development of adaptive methodologies to address uncertainties and data limitations in investigating extrajudicial killings and forced disappearances may assist in overcoming shortcomings. The findings further highlight the need for investigatory systems that learn, adapt and recognise uncertainty, especially in the face of limited data where decision-making can lead to poor outcomes. Extrajudicial killings and forced disappearances often occur in chaotic and unpredictable contexts, characterised by extreme uncertainty, necessitating a cautious approach in forensic investigations. As summarised in Table 1., the results of this review highlight significant practical challenges faced by forensic science and medicine. While these themes often intersect, their distinctive characteristics merit further discussion.

Despite extensive research covering topics such as mass graves [71–75], post-conflict case studies [72, 76], experimental research, [77–80] best burial practices [81], the recovery of war dead [82, 83], and deaths related to migration [22, 84–87], literature addressing unlawful killings such as extrajudicial executions and forced disappearances remains limited. While the parameters of this review were for specific literature from a forensic medicine/science perspective, only 44 publications were found for discussion. Given the enormity of the problem, significant challenges in accessing or potentially identifying the existence of legal, operational and statistical data [8] result in gaps in the empirical literature on the subject.

As noted in the introduction, these types of killings can often follow a sequence of events—capture, detention, and disposal [3]. Signs of healing or healed fractures may indicate prolonged exposure to torture and abuse, providing critical insights into the sequence of events [4–6]. However, the confirmation of torture and abuse is challenging when analysing remains several months or years after the physical trauma has occurred [64]. While antemortem trauma can be indicative of chronic maltreatment [62] and attributable to the time victims were detained for injuries to heal [63], the interval between death and recovery can span years. Skeletal changes occur after 6 months if medical treatment is not provided, causing the dislocated joint surfaces to misalign, leading to degenerative joint disease, new bone formation on the joint surface or secondary or false joints [65]. Residual deformities are observable in radiographs (i.e. opacification of maxillary sinuses due to beatings that can cause sinusitis) when detention and beatings are prolonged [47]. However, skeletal lesions may not fully account for the traumatic impacts that occur to a body, given bones are resistant to impact [39]. This is evidenced in studies where differences in the body's condition in estimating cause/manner of death were between 83–79% but declined to 40% when less than half of the body was available and when decomposition was a factor. The percentage of the body recovered significantly impacted the presence or absence of trauma, where complete remains were 10.4% more likely to show signs of trauma [55]. While it may be possible with recently skeletonised cases to see discolouration around trauma defects, indicating increased blood flow and healing [88], in many instances, years or decades may pass before analysis and autopsy. For example, Palmatoria (torture technique where the anterior tibia is repeatedly struck), where the anterior cortex of the tibia is thickened by a periosteal reaction due to subperiosteal haemorrhage and hematoma [47]. A periostitis may last weeks or years and show hidden endosteal fractures. However, these more sophisticated imaging modalities, such as computed tomography, [47] may not be available in contexts where abuses have occurred. Furthermore, taphonomic changes can erase traces of injuries, whereas fatal injuries that affect soft tissues leave no traces on skeletal tissue [40].

Further complicating the investigation of extrajudicial killings and forced disappearances is when remains suspected of belonging to victims are severely commingled [38], missing, or degraded, making it challenging to evaluate features [50] suggestive of the dynamics of death [52, 53]. If remains are commingled, not only can identification be hindered, but the cause and manner of death cannot be thoroughly evaluated until remains have been segregated into complete individuals [89]. Victims may seldom be killed and buried in the exact location [35], and delays in the investigation can result in remains becoming skeletonised, reducing the ability to assess injuries or identify the victims [9, 10]. This adds another dimension to the problem. Identification, a legal determinate based on the scientific matching of information derived from missing persons’ records with information obtained from unidentified human remains [90], is further complicated by contexts featuring social and political instability. As demonstrated in Spain during the Civil War, even under conditions of civil conflict, medicolegal death investigations may still be conducted, albeit to a lower standard. Such a compromised investigation may suggest a systemic shift towards extrajudicial tactics [37]. In such contexts, extrajudicial killings and forced disappearances may be commonplace and compromised resources and logistic constraints may degrade the quality of antemortem records [56, 57], exacerbating technical issues [58, 68]. In older cases, historical records may be helpful if available; however, without documents to link to victims, it may be challenging to provide culpability and victim identification [91].

A key question is how research can inform practice in establishing that the remains being analysed were the result of unlawful killings. Limited available data may still be helpful across jurisdictions to support studies that track change over time alongside methodologies that acknowledge the uncertainties in forensic data analysis. While these patterns can provide critical insights into the sequence leading to death, developing epidemiological frameworks for the interpretation of skeletal trauma [55, 69] can assess data trends to distinguish trauma indicative of these types of killings. This has been done in places such as Colombia, where extrajudicial killings and forced disappearances can occur within the context of war, where such deaths differ distinctively differences from combat fatalities [36]. Patterns of trauma, such as repeated injuries to specific regions of the body, can help differentiate lawful combat-related deaths from these types of unlawful killings. This can be useful as it provides a basis to statistically compare differences between combat deaths and those where victims were explicitly targeted and where aspects such as torture and abuse have occurred. Such frameworks may also inform immediate forensic needs, support long-term community healing, and guide surveillance systems that monitor and report on incidents to enhance practice, research and policy. The development of bottom-up community-driven mechanisms that balance legal requirements with sociocultural sensitivities to ensure dignity for the deceased while promoting effective investigations and risk mitigation and prevention strategies can be derived from this research. For example, examining pathologies indicative of health status among individuals killed at Srebrenica provided policymakers with risk factors useful in their assessments of future genocide prevention [51]. Similar approaches may apply to potential victims of extrajudicial killings and forced disappearances. While the works discussed above are helpful, there is a significant disconnect between theoretical perspectives and their practical implementation, as evidenced by the limited empirical examples. Although theoretical research often proposes aspirational best-case scenarios, evaluating their adaptability across diverse socio-political contexts is challenging. These cases frequently occur in chaotic and unpredictable contexts that resist straightforward theoretical application. As a result, assessing their effectiveness in impacting medicolegal practice locally remains challenging, particularly in the absence of quality data and the ability to observe their implementation.

## Study limitations

Challenges with this topic stem from the fact that the types of killings discussed in this review generally involve a sequence of events encompassing the capture, detention and eventual killing of an individual for which forensic evidence is vital in determining the manner and cause of death. It is an issue of information asymmetry, acknowledging that the available data cannot account for all aspects of the phenomena, which affects the reliability and conclusions derived from the data, thus impacting how unlawful killings are understood. This task is complicated as many factors may affect the analysis and interpretation. The complexities inherent in these types of deaths are reflected in the lack of specific empirical literature on the subject. This review investigated forensic literature to assess the present status of forensic medical research concerning extrajudicial killings and forced disappearances. Extrajudicial killings are complex issues. Although the authors recognise the broad legal, social, and political perspectives on this subject, the focus primarily centres on individuals who are directly targeted, tortured, and ultimately killed. Lastly, the inclusion and exclusion criteria may have missed articles that could have been useful for the review.

## Conclusions

A theme that reoccurs throughout this review is that there is a significant deficit in access to and effective investigation of the remains of those killed unlawfully. Furthermore, empirical research in forensic science that can inform practice is significantly underrepresented. These types of deaths pose significant complexities due to legal, operational, and statistical challenges in not only accessing remains but also in the limited capacity to research the subject. Access to remains for post-mortem examination is undoubtedly a critical factor in the limited scope of this topic within the forensic literature. However, as discussed in this review, the existing literature that investigates this topic provides valuable insight into this complex area and serves as a foundation for further exploration. Forensic methodologies should withstand the uncertainties and challenges accompanying these investigations and adapt and improve through exposure to such stressors. Adaptability to uncertainty, as exemplified in these contexts, can provide opportunities for research and innovation into these tragic events. This review highlights the potential for enhanced data collection and modelling. Specifically, such advancements may significantly improve the impact of interventions aimed at reducing the risk of unlawful killings. Systems that benefit from variability and complexity – such as adaptive forensic protocols that will enhance under stress – have the potential to turn obstacles into sources of success and resilience. Given the limitations in knowledge inherent in these types of forensic investigations, flexibility and adaptability are crucial. Providing updated predictions as new evidence becomes available can refine risk predictions in real-time, thus aiding in better decision-making to address these deaths' uncertainties. Such research can play a vital role aid in mitigating the impunity often surrounding these types of deaths, which profoundly affect families and communities, leaving them without recourse, resolution, or the means of obtaining justice.

## Data Availability

NA.

## Code availability

NA.
